# Bacterial contamination and prevalence of antibiotic-resistant *Escherichia coli* and *Salmonella* isolated from raw pork and chicken meat from slaughterhouses and retail markets in Kenya

**DOI:** 10.14202/vetworld.2026.1867-1887

**Published:** 2026-05-10

**Authors:** Metrine Namalwa Nyanja, Christine Minoo Mbindyo, Daniel Wambua Wanja, Jackson Ombui, Getrude Shepelo Peter, Felix M Kibegwa, George C Gitao, Charles Drago Kato

**Affiliations:** 1 Department of Public Health Pharmacology and Toxicology Pathology, Faculty of Veterinary Medicine, University of Nairobi, Nairobi, Kenya; 2 Department of Veterinary Pathology, Microbiology and Parasitology, Faculty of Veterinary Medicine, University of Nairobi, Nairobi, Kenya; 3 Department of Veterinary Pathology, Microbiology and Parasitology, Faculty of Veterinary Medicine and Surgery, Egerton University, Njoro-Egerton, Kenya; 4 Department of Clinical Studies, Faculty of Veterinary Medicine, University of Nairobi, Nairobi, Kenya; 5 Department of Animal Production, Faculty of Veterinary Medicine, University of Nairobi, Nairobi, Kenya; 6 School of Biosecurity, Biotechnical and Laboratory Sciences, College of Veterinary Medicine, Animal Resources and Biosecurity, Makerere University, Kampala, Uganda

**Keywords:** antimicrobial resistance, *Escherichia coli*, food safety, Kenya, meat contamination, multidrug resistance, pork and chicken, *Salmonella*

## Abstract

**Background and Aim:**

Foodborne diseases caused by bacterial contamination of meat remain a major global public health concern, particularly in low- and middle-income countries. *Escherichia coli* and *Salmonella* are among the most important zoonotic pathogens associated with raw meat, and their increasing antimicrobial resistance (AMR) further complicates treatment and control. In Kenya, limited data exist on bacterial contamination and resistance patterns along the meat value chain, especially in peri-urban and rural settings. This study aimed to quantify bacterial contamination levels and determine the prevalence and AMR profiles of *E. coli* and *Salmonella* species isolated from raw chicken meat and pork collected from slaughterhouses and retail outlets in Murang’a County, Kenya.

**Materials and Methods:**

A cross-sectional study was conducted between November 2024 and June 2025. A total of 320 meat samples (105 chicken and 215 pork) were collected from slaughterhouses, butcher shops, supermarkets, and public wet markets. Total viable counts (TVC) were determined using standard plate count methods. Isolation and identification of *E. coli* and *Salmonella* species were performed using conventional culture techniques and confirmed by matrix-assisted laser desorption/ionization–time-of-flight mass spectrometry. Antimicrobial susceptibility testing was conducted using the Kirby–Bauer disk diffusion method following Clinical and Laboratory Standards Institute guidelines. Multiple antibiotic resistance (MAR) indices and resistance phenotypes were also determined.

**Results:**

High levels of bacterial contamination were observed, with mean TVC exceeding recommended limits (6.45 log_10_ CFU/cm² for chicken and 6.39 log_10_ CFU/cm² for pork). Overall prevalence of *E. coli* was 61.6%, while *Salmonella* species were detected in 7.2% of samples. AMR was widespread, particularly in *E. coli*, with high resistance to ampicillin (95.3%), amoxicillin–clavulanate (78.7%), gentamicin (72.7%), and tetracycline (72.0%). Multidrug resistance was detected in 87.3% of *E. coli* isolates, with 16.0% classified as extensively drug-resistant and one isolate identified as possible pandrug-resistant. All isolates exhibited MAR indices ≥0.1, indicating exposure to high-risk antimicrobial environments. *Salmonella* isolates showed the highest resistance to ciprofloxacin (86.4%) and lower multidrug resistance (13.6%).

**Conclusion:**

The study demonstrates substantial bacterial contamination and high prevalence of antimicrobial-resistant pathogens in raw meat along the Kenyan meat value chain. These findings highlight the urgent need for strengthened hygiene practices, improved food safety regulation, and enhanced AMR surveillance within a One Health framework to mitigate public health risks.

## INTRODUCTION

Global meat consumption continues to rise and is projected to reach 460–570 million tons annually by 2050 [1]. In Kenya, national per capita meat consumption has grown rapidly, with chicken and pork showing the fastest growth, largely driven by urbanization, increasing income, and population growth [2–4]. Approximately 92.6% of the population frequently consumes chicken, indicating its popularity [2]. Although pork consumption is still comparatively low, at about 0.4 kg per person annually, it has grown significantly over the last decade [4, 5]. Meat is a major source of protein [6]; however, its nutrient-rich nature provides a favorable environment for pathogenic bacterial growth and contamination [7].

Unsafe food, including meat contaminated with bacteria and associated antimicrobial resistance (AMR), poses a significant public health threat worldwide, and more so in Africa, due to limited food safety systems [8, 9]. According to the World Health Organization (WHO), foodborne diseases remain a major global public health concern, causing an estimated 600 million illnesses, 420,000 deaths, and approximately 33 million disability-adjusted life years annually worldwide [10, 11]. Although these estimates were originally derived from the WHO Global Burden of Foodborne Diseases study using 2010 data, they continue to represent the best available and most widely cited global burden figures, and WHO has consistently reaffirmed their relevance in subsequent reports and updates. Africa is estimated to have the highest burden of foodborne diseases at (2,460 disability-adjusted life years per 100,000 population) compared to all regions analyzed [12, 13]. The main cause of foodborne diseases in humans is the consumption of contaminated foods by bacteria and toxins, which can occur at any stage of the food production, delivery, and consumption chain [9, 12].

Zoonotic bacteria, such as *Salmonella enterica* serovars and *E. coli* pathotypes, are the major causes of foodborne diseases, primarily manifesting as diarrhea and enteritis in humans worldwide and in Africa [9, 14–16]. *Salmonella* spp. remains among the most important foodborne pathogens globally, with a substantial disease burden in low- and middle-income countries. In recognition of the increasing threat posed by antimicrobial resistance (AMR), the WHO included fluoroquinolone-resistant *Salmonella Typhi* and fluoroquinolone-resistant non-typhoidal *Salmonella* in the high-priority tier of the WHO Bacterial Priority Pathogens List 2024. This classification highlights the public health importance of resistant *Salmonella* infections and the growing limitations in treatment options. The inclusion of *Salmonella* in this study is therefore justified, given its clinical relevance and its role in AMR surveillance within a One Health framework.

Most *E. coli* strains live harmlessly in human and animal intestines, but some have acquired virulence factors that enable them to cause disease, making them important zoonotic foodborne pathogens. Diarrheagenic *E. coli* pathotypes can cause many sporadic cases and foodborne outbreaks; they pose a major public health threat. In Kenya, studies have shown that human enteric fevers caused by *Salmonella* species and *E. coli* are endemic, particularly in urban areas, with children under 5 years being the most vulnerable group [15, 17, 18]. However, there are generally few studies on foodborne diseases in peri-urban and rural communities, which limits food safety interventions [14, 19, 20]. This study targeted generic *E. coli* as an indicator of fecal contamination and hygiene status, rather than specific diarrheagenic pathotypes.

The rising emergence of multidrug resistance among foodborne pathogens, including *Salmonella* and *E. coli*, represents a significant global public health issue [21, 22]. Food-producing animals, especially chickens and pigs and their meat are the major reservoirs of multidrug-resistant *Salmonella* and *E. coli* bacteria [23, 24]. Antimicrobial-resistant bacteria and resistance genes can easily be transferred to humans through direct contact and consumption of contaminated food, including meat [14, 15]. The unregulated use of antimicrobials in food animals for growth enhancement or prophylactic purposes heightens the public health risk of zoonotic multidrug-resistant *Salmonella* and *E. coli*, especially in developing countries like Kenya [22, 23, 25, 26]. These resistant strains may remain in animal-derived products, such as meat, milk, and eggs, posing a risk to human and animal health [23, 24, 27].

Additionally, slaughter facilities and retail outlets in Kenya are potential hotspots for the dissemination of antimicrobial-resistant *E. coli* and *Salmonella* along the meat value chain [28–30]. Previous studies have reported the presence of antimicrobial-resistant *E. coli* and *Salmonella* spp. in meat, retail outlets, and slaughterhouse environments, including resistance to critically important antimicrobials not commonly used in veterinary practice, signifying a major public health risk [28, 30]. Moreover, inadequate enforcement of food safety regulations in slaughter facilities and meat retail outlets in Kenya increases the risk of meat contamination with\ antimicrobial-resistant bacteria [29, 31, 32]. Bacterial contamination risks can persist throughout production from slaughter to market, affecting the meat quality and leading to human infections [33, 34].

Further, in sub-Saharan Africa, including Kenya, informal slaughter practices, unhygienic environments, poor hygiene standards of slaughter equipment, and poor personal hygiene practices in retail outlets remain major contributors to microbial contamination of meat products [33, 35, 36]. Workers in those slaughter facilities and retail markets may acquire antimicrobial-resistant bacteria through direct contact during meat handling, as many lack proper protective clothing [29]. Therefore, there is an urgent need for continuous AMR surveillance to determine the risk of consumer exposure to multidrug-resistant enteric bacteria originating from foods [37].

To date in Kenya, several studies have reported the high presence of antimicrobial-resistant bacteria in food animals, including chicken and pigs [17, 18, 38, 39]. However, studies on the food safety and antimicrobial-resistant bacteria contaminating the meat along the value chain in peri-urban and rural communities in Kenya are limited [30, 31]. This is despite the increasing consumption of chicken meat and pork in these communities, driven by industrialization and rising household purchasing power [34]. In addition, reports of food-linked diarrhea outbreaks in those regions are frequent [14, 40].

Consistent with findings from previous studies [23, 41], this study assessed bacterial contamination levels in raw chicken meat and pork obtained from slaughterhouses and retail outlets in Murang’a County, Central Kenya, and determined the prevalence of antimicrobial-resistant *E. coli* and *Salmonella* species. These findings reinforce existing evidence on the burden of bacterial contamination and AMR associated with raw meats and provide additional, location-specific data that may inform the development of targeted interventions to strengthen food safety systems in Kenya.

Despite growing evidence on bacterial contamination and AMR in food animals in Kenya, there remains a critical lack of comprehensive, value-chain–based studies that simultaneously evaluate contamination levels, pathogen prevalence, and resistance profiles across both slaughterhouses and retail outlets, particularly in peri-urban and rural settings. Most existing studies have focused on either specific pathogens, limited geographic regions, or isolated points within the meat production and distribution chain, thereby failing to provide an integrated understanding of contamination dynamics from slaughter to the consumer level. Furthermore, limited data exist on the comparative burden of antimicrobial-resistant *E. coli* and *Salmonella* in pork and chicken within these underrepresented regions, where food safety infrastructure and regulatory enforcement are often inadequate. This gap limits the ability to design targeted, evidence-based interventions to improve meat hygiene and mitigate AMR risks in the Kenyan context.

Therefore, this study aimed to comprehensively assess bacterial contamination levels and to determine the prevalence and AMR profiles of *E. coli* and *Salmonella* species isolated from raw chicken meat and pork across multiple points along the meat value chain, including slaughterhouses and retail outlets in Murang’a County, Kenya. Specifically, the study sought to (i) quantify total bacterial load in meat samples as an indicator of hygienic quality, (ii) isolate and identify *E. coli* and *Salmonella* species, (iii) evaluate antimicrobial susceptibility patterns and resistance phenotypes, and (iv) characterize multidrug resistance and associated risk indicators. By integrating microbiological and resistance data across different value-chain stages, this study provides critical insights into contamination pathways and AMR dissemination, thereby supporting the development of targeted food safety and antimicrobial stewardship interventions within a One Health framework.

## MATERIALS AND METHODS

### Ethical approval

Ethical approval for this study was obtained from the Biosafety, Animal Use, and Ethics Committee of the Faculty of Veterinary Medicine, University of Nairobi, Nairobi, Kenya (Approval No. FVM BAUEC/2024/579). Additional administrative permission to conduct the study was granted by the Veterinary Department of Murang’a County, Kenya. The study involved collection of raw chicken meat and pork samples intended for human consumption from slaughterhouses, butcher shops, supermarkets, and public wet markets, and therefore did not involve direct experimentation on live animals. Samples were obtained only from carcasses and meat products that were already prepared for commercial sale or slaughtered as part of routine commercial operations independent of the study.

Permission to collect samples was obtained from owners, managers, and/or operators of the participating establishments before sampling. Verbal informed consent was obtained from meat vendors and outlet operators after explaining the purpose of the study, the sampling procedures, and the intended use of the data generated. Participation was entirely voluntary, and no personal identifiers of vendors, workers, or establishments were disclosed in the study records or publication. Confidentiality of information obtained during data collection was maintained throughout the study.

All laboratory procedures were conducted in accordance with institutional biosafety requirements under Biosafety Level 2 conditions in the Department of Public Health, Pharmacology and Toxicology, University of Nairobi. Standard microbiological safety procedures were followed during sample handling, bacterial isolation, identification, storage, and antimicrobial susceptibility testing to minimize risks to personnel and the environment. Biological materials and laboratory waste were handled, decontaminated, and disposed of according to approved institutional biosafety and waste management protocols.

Because the study did not involve live animal handling, invasive procedures, animal restraint, or experimental infection, no additional animal welfare intervention was required. The work was observational and laboratory-based, with all meat samples collected from routine commercial value-chain operations.

### Study period and area

**Figure 1:**
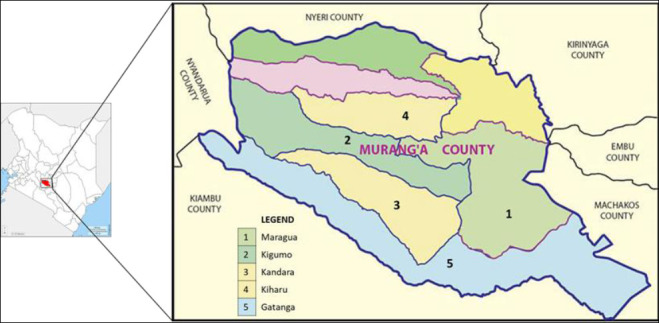
Map of Kenya showing the location of Murang’a County (shaded red) and the location of study sites, including Maragua, Kigumo, Kandara, Kiharu, and Gatanga sub-counties within the county (numbered 1-5). Map generated using Google Maps API.

The study was conducted from November 2024 to June 2025 in Murang’a County, located in Central Kenya, approximately 85 km northeast of Nairobi, between latitudes 0° 34’ S and 1° 7’ S and longitudes 36° E and 37°27’ E (Figure 1). Murang’a County borders Nyeri to the north, Nyandarua to the west, Kiambu to the south, and Kirinyaga to the east, and covers 2,524.2 km². Administratively, the county is divided into eight sub-counties. However, sampling was carried out in only five sub-counties: Maragua, Kigumo, Kandara, Kiharu, and Gatanga.

These study sites were purposively selected because they contain major towns with many chicken and pork retail outlets, as well as several formal and informal slaughter points. According to the 2019 census, the county had 1,056,640 residents, with a population density of 419 persons/km². Agriculture is the main economic activity and supports 80% of households, with poultry and pig farming emerging as important agribusinesses [42].

### Study design

A cross-sectional study was conducted between November 2024 and June 2025 in five sub-counties of Murang’a County.

### Study population and eligibility criteria

The target population for this study comprised all raw chicken meat and pork intended for human consumption in Murang’a County, Kenya, including products obtained from retail outlets such as butcher shops and supermarkets, public wet markets, and slaughterhouses.

Meat samples eligible for inclusion in this study were raw chicken and pork intended for human consumption in Murang’a County, collected from slaughterhouses, public wet markets, and retail outlets, including butcher shops and supermarkets. Only fresh, intact cuts of meat that were free from visible signs of spoilage were included. Cooked, heavily processed (canned or minced), or long-frozen products, as well as samples showing overt spoilage or contamination, or outlets selling other types of meat, were excluded. In addition, samples from outlets or suppliers who declined to provide informed consent for participation were not included in the study.

### Sample size determination and sampling strategy

Among the two bacterial isolates, *Salmonella* is relatively uncommon; therefore, the anticipated prevalence in pork and chicken meat was set at 16.4% and 6%, respectively, based on studies by *Ejeta et al*. [43] and *Elbayoumi et al*. [44]. Sample size was calculated using Naing’s formula: n = (Z²P(1-P))/d², where n is the required sample size, Z is the Z-statistic at 95% confidence (1.96), P is the expected prevalence, and d is the absolute precision (5%, or 0.05) [45]. The minimum calculated sample sizes were 211 for pork and 87 for chicken meat. These were increased to 215 and 105, respectively, to account for potential sample attrition and to improve precision.

Murang’a County had two registered slaughterhouses handling pigs and 24 unlicensed public wet markets for chicken; consequently, a census sampling strategy was employed at this level. Each slaughterhouse and/or public wet market was sampled twice at four-week intervals to account for short-term temporal variability in bacterial contamination. During each sampling visit, raw chicken meat and pork specimens were collected aseptically from freshly slaughtered carcasses. Repeated sampling was conducted to enhance the representativeness of the data and to reduce potential bias arising from day-to-day operational variability.

Retail outlets, comprising butcher shops and supermarkets, were selected by simple random sampling, with the number of outlets per sub-county determined proportionally to the total number of outlets in each sub-county. At each selected outlet, one raw chicken meat sample and/or one pork sample, depending on availability, was collected aseptically. Samples were allocated between slaughterhouses and retail outlets, with a greater proportion collected from retail outlets to reflect higher consumer exposure.

### Sample collection

A total of 320 samples of raw chicken and pork were randomly collected from the study site, as shown in Table 1. Out of the 320 samples, 105 were chicken meat, and 215 were pork, sourced from slaughterhouses (n = 51), butcher shops (n = 208), supermarkets (n = 13), and public wet markets (n = 48). The detailed distribution of samples across the meat value chain sources in the various sub-counties is shown in Table 1. The sample distribution was based on the availability of an operational sampling point at the time of the study.

**Table 1:** Distribution of raw chicken meat and pork samples collected from different slaughterhouses and retail markets in various sub-counties in Murang’a County, Kenya.

Sampling point	Category	Location	Pork (n)	Chicken (n)	Total (n)
Slaughterhouses	Sub-county	Maragwa	26	–	26
		Kandara	25	–	25
	Subtotal		51	–	51
Butcher shops	Sub-county	Maragwa	73	30	103
		Kiharu	45	14	59
		Kandara	29	–	29
		Kigumo	9	–	9
		Gatanga	8	–	8
	Subtotal		164	44	208
Supermarkets	Sub-county	Maragwa	–	3	3
		Kiharu	–	8	8
		Kandara	–	2	2
	Subtotal		0	13	13
Public wet markets	Sub-county	Maragwa	–	31	31
		Kiharu	–	1	1
		Kandara	–	16	16
	Subtotal		0	48	48
Overall total			215	105	320

**Figure 2:**
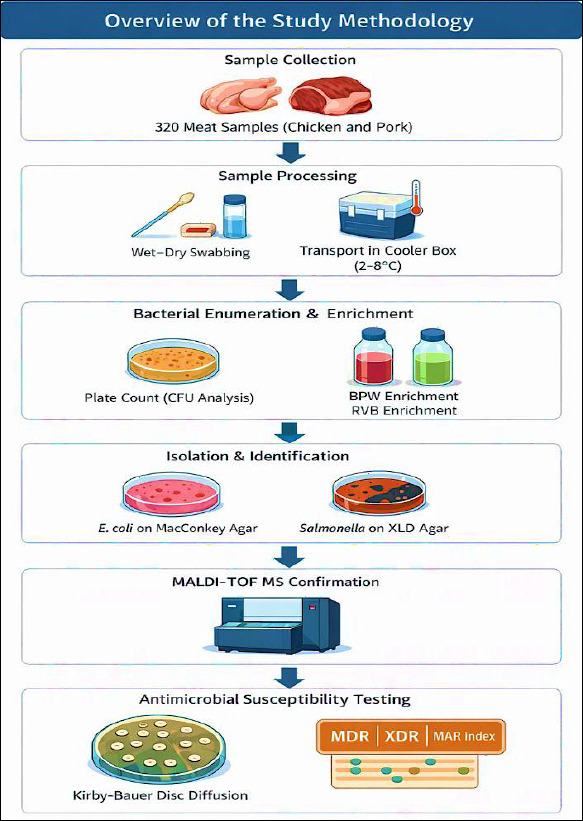
Schematic overview of the study design and methodological workflow.

The meat samples were purchased either as whole chickens or as 100 g portions of chopped chicken meat and 100 g of dressed pork, obtained from any part of the carcass at the owner’s discretion. Each sample was placed in a sterile zip-lock bag, labeled, and transported in cooler boxes containing frozen gel packs to maintain a 2°C–8°C cold chain to the Microbiology Laboratory (Department of Public Health, Pharmacology and Toxicology, University of Nairobi) for analysis within 4 h of collection. The overall study workflow is summarized in Figure 2.

### Sample processing

The samples were removed from the cooler boxes and allowed to thaw at room temperature (24–26°C) before processing. Sterile cotton swabs were used following a wet–dry swabbing technique to sample a standardized 100 cm² area of each meat specimen. For the wet swab, the swab was immersed in phosphate-buffered saline and used to swab the area in two perpendicular directions for 20 s. This was followed by a dry swab of the same area to maximize microorganism recovery, as described by *Berhanu et al*. [46]. The swabs were then suspended in 9 mL phosphate-buffered saline and vortexed to form the stock solution (10^0^). The stock bacterial suspension (10^0^) was initially used for bacterial enumeration, while the remaining suspension was used to isolate *E. coli* and *Salmonella* species.

### Enumeration of bacteria (total viable counts [TVC]) in meat samples

For bacterial enumeration, the stock bacterial suspension (10^0^) was serially diluted in 9 mL of phosphate-buffered saline to obtain 10-fold dilutions. An aliquot (0.1 mL) of the undiluted suspension (10^0^), and serial dilutions (10^3^, 10^4^ and 10^5^), were aseptically plated onto Plate Count agar (HiMedia Laboratories Pvt. Ltd., Mumbai, India). The inoculum was evenly spread using a sterile glass rod, and the plates were then incubated aerobically at 37°C for 24 h. Samples were plated in duplicate to ensure the reliability and reproducibility of results. The mean colony counts were used for analysis. Colony-forming units were enumerated and interpreted according to the Commission of the European Communities guidelines [47]. Aerobic plate counts were classified as (i) acceptable, if counts were ≤ 1 × 10^4^ colony-forming units/cm² (4 log); (ii) marginal, if counts were ≤ 1 × 10^5^ colony-forming units/cm² (5.0 log); and (iii) unacceptable, if counts were > 1 × 10^5^ colony-forming units/cm² (5.0 log). The classification thresholds were based on established process hygiene benchmarks and international guidance used in food microbiology, including European Union/Food and Agriculture Organization recommendations for interpreting microbial load in meat products.

### Isolation and identification of *E. coli* and *Salmonella* species

Detection and identification of *E. coli* and *Salmonella* species were performed according to International Organization for Standardization (ISO 6579-1:2017) [48] and *Markey et al*. [49]. Following bacterial enumeration, 1 mL of the remaining stock solution (10^−1^) from each meat sample was aseptically inoculated into 9 mL of sterile buffered peptone water (Oxoid Ltd., Basingstoke, UK) for non-selective enrichment, and another 1 mL was inoculated into 9 mL of Rappaport–Vassiliadis broth (Oxoid Ltd., Basingstoke, UK) for selective enrichment of *Salmonella* spp. The inoculated broths were vortexed for 10–15 s and incubated at 37°C for 24 h before further processing.

Following enrichment, a loopful of buffered peptone water broth was aseptically streaked onto MacConkey agar (Oxoid Ltd., Basingstoke, UK) for the isolation of *E. coli*, while Rappaport–Vassiliadis broth was streaked onto Xylose Lysine Deoxycholate agar (Oxoid Ltd., Basingstoke, UK) for the isolation of *Salmonella* spp. The plates were then incubated at 37°C for 18–24 h and examined for characteristic colonies. Quality control included parallel processing of positive and negative controls: *Salmonella enterica* ATCC 14028 for *Salmonella* detection, *E. coli* ATCC 25922 for lactose-fermenting Enterobacteriaceae, and sterile buffered peptone water as a negative control for contamination. Presumptive identification was based on colony morphology: *E. coli* formed small lactose-fermenting (pink) colonies on MacConkey agar, and *Salmonella* spp. produced red colonies with black centers on Xylose Lysine Deoxycholate agar. Representative colonies were sub-cultured and confirmed as Gram-negative rods by Gram staining, followed by standard biochemical tests. Catalase and oxidase tests verified Enterobacteriaceae characteristics (catalase-positive, oxidase-negative). Indole, methyl red, Voges–Proskauer, and citrate utilization tests differentiated species: *E. coli* is indole-positive, methyl red–positive, Voges–Proskauer–negative, and citrate-negative, whereas *Salmonella* spp. are indole-negative, methyl red–positive, Voges–Proskauer–negative, and citrate-positive. Urease testing excluded urease-positive Enterobacteriaceae. Triple sugar iron agar was used to assess carbohydrate fermentation and H_2_S production: *Salmonella* spp. typically shows an alkaline slant/acid butt with H_2_S, whereas *E. coli* shows an acid slant/acid butt without H_2_S.

### Matrix-assisted laser desorption/ionization–time of flight mass spectrometry (MALDI-TOF MS) confirmation of bacterial isolates and spectral validation

Confirmation of identity was performed using MALDI-TOF MS (Bruker Daltonics GmbH, Bremen, Germany). Standard Bruker interpretative criteria were applied [50]. Briefly, scores of ≥2.0 were accepted for species assignment and scores of ≥1.7 but <2.0 for identification to the genus level. Briefly, isolates were identified by direct colony transfer following the manufacturer’s instructions. A fresh (18–24 h) single colony was applied to the stainless-steel target plate and overlaid with 1 μL of α-cyano-4-hydroxycinnamic acid matrix. Isolates with initial scores below acceptance thresholds underwent an extended ethanol–formic acid extraction to improve spectral quality. Each isolate was analyzed in duplicate, and the highest score was used for final identification. Spectra with scores <1.7 were considered unreliable identification; these isolates were re-spotted, re-analyzed, and, if needed, re-cultured and re-tested. Only isolates achieving acceptable Bruker scores after repeat analysis were included in downstream interpretation. Quality control of the spectra was ensured by analyzing reference strains *E. coli* ATCC 25922, *Pseudomonas aeruginosa* ATCC 27853, and *Salmonella enterica* ATCC 14028.

All confirmed isolates in pure culture were preserved at −80°C in skimmed milk (Oxoid Ltd., Basingstoke, UK) for subsequent analysis. Recovery was confirmed by thawing a subset of samples and assessing growth on the respective media.

### Antimicrobial susceptibility testing

Of the 197 confirmed *E. coli* isolates, 150 were subjected to antimicrobial susceptibility testing due to resource and reagent limitations, with selection ensuring representation across sampling locations and sample types. For *Salmonella* spp., 22 of the 23 confirmed isolates were successfully revived and included in antimicrobial susceptibility testing, while one isolate failed to recover despite repeated subculture attempts. Antimicrobial susceptibility testing was subsequently performed using the Kirby–Bauer disk diffusion method in accordance with the Clinical and Laboratory Standards Institute [Clinical and Laboratory Standards Institute M100, 34th Edition (2025)] guidelines [51]. Briefly, freshly cultured isolates grown on blood agar were emulsified in phosphate-buffered saline, and the suspension was adjusted to a 0.5 McFarland turbidity standard. The standardized bacterial suspension was then uniformly swabbed onto Mueller-Hinton agar (Oxoid Ltd., Basingstoke, UK) plates and allowed to dry before placement of antibiotic disks. The antibiotic panel tested on the isolates was drawn from nine antimicrobial classes as follows: penicillins [10 μg ampicillin (AMP), and 30/10 μg amoxicillin-clavulanic acid (AMC)], cephalosporin [30 μg ceftriaxone (CRO) and 30 μg ceftazidime (CAZ)], carbapenem [10 μg imipenem (IMP)], macrolides [15 μg azithromycin (AZI)], aminoglycosides [10 μg gentamicin (CN)], tetracyclines [30 μg tetracycline (TET)], fluoroquinolone [5 μg ciprofloxacin (CIP)], sulphonamide [1.25/23.75 μg sulphamethoxazole/trimethoprim (STX)], and amphenicols [30 μg chloramphenicol (C)]. These antimicrobial agents were selected because the WHO classifies them as critically or highly important for human medicine, and they are widely used for treatment and prevention in veterinary practice in Kenya. The plates were then incubated aerobically at 37°C for 18 h. Thereafter, each inhibition zone diameter was accurately measured and then interpreted according to Clinical and Laboratory Standards Institute breakpoints [51]. *E. coli* ATCC® 25922, *Staphylococcus aureus* ATCC® 25923, and *Pseudomonas aeruginosa* ATCC® 27853 were included as quality control strains.

The MDR isolates were defined as those resistant to one or more antimicrobials in at least three different classes, while isolates resistant to one agent from all antimicrobial categories except one or two categories were termed extensively drug-resistant (XDR). Meanwhile, isolates resistant to all agents in all antimicrobial categories were described as pan-drug-resistant (PDR) [52]. The MAR indices of the isolates were determined by the following equation: MAR = a/b, where ‘a ’ represents the number of antibiotics to which the isolate was resistant and ‘b’ represents the total number of antibiotics tested [52]. In this study, ‘b’ was 10 for *E. coli* and 8 for *Salmonella* isolates. A MAR index > 0.2 was interpreted as indicative of isolates originating from high-risk environments where antibiotics are frequently or indiscriminately used [52].

### Statistical analysis

Data entry and management were performed using Microsoft Excel. Data analysis was conducted using IBM Statistical Package for the Social Sciences (SPSS; IBM Corp., Armonk, NY, USA), version 31. Prevalence was calculated as the proportion of positive samples among those tested. Descriptive statistics were used to summarize frequencies and proportions. TVC, expressed as log_10_ colony-forming units/cm², were summarized using descriptive statistics expressed as mean ± standard deviation. Before inferential analysis, the distribution of TVC data was assessed for normality using graphical methods and summary measures. Because the data were non-normally distributed and included unequal sample sizes across value-chain sources, a non-parametric approach was adopted. Differences in TVC among butcher shops, supermarkets, and public wet markets were evaluated using the Kruskal–Wallis H test. Where the Kruskal–Wallis test indicated statistically significant differences (p < 0.05), post hoc pairwise comparisons were performed using Dunn’s test with Bonferroni correction to control for multiple comparisons. Chi-square was used to compare prevalences of *E. coli* and *Salmonella* in meat types and value-chain sources. For statistical analysis, 95% confidence intervals (95% CIs) for proportions were calculated using both the normal approximation (Wald method) and the exact Clopper–Pearson binomial method (applied in cases of small sample sizes), and p < 0.05 was considered statistically significant.

### Quality control

All procedures were standardized to ensure accuracy and reliability. Field and laboratory blanks (sterile phosphate-buffered saline, pH 7.2) were processed alongside meat samples to monitor contamination. Bacterial identification by MALDI-TOF MS was quality-controlled using reference strains (*E. coli* ATCC 25922, *P. aeruginosa* ATCC 27853, and *S. enterica* ATCC 14028). Antimicrobial susceptibility testing was performed according to established guidelines, with routine quality control strains (*E. coli* ATCC 25922, *S. aureus* ATCC 25923, and *P. aeruginosa* ATCC 27853); zone diameters were verified within recommended ranges. Equipment was regularly calibrated, and antimicrobial disks were stored according to manufacturer instructions.

## RESULTS

### Enumeration of TVC in chicken meat samples

**Figure 3:**
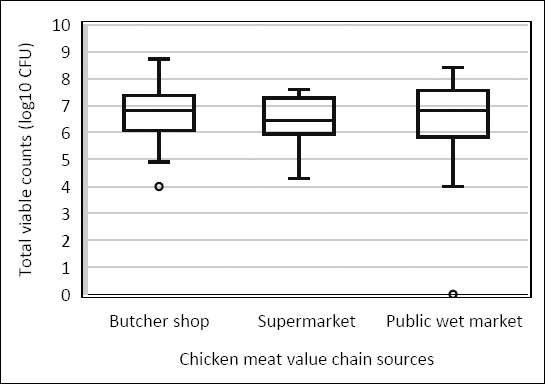
Distribution of total viable bacterial counts (log_10_ CFU/cm²) in raw chicken meat obtained from butcheries, supermarkets, and public wet markets in Murang’a County, Kenya. The central line represents the median, boxes indicate the interquartile range, and whiskers represent the minimum and maximum values (n = 105; outliers are represented as circles).

A total of 105 chicken meat samples were analyzed, including 44 from the butcher shops, 13 from the supermarkets, and 48 from public wet markets. The mean TVC of chicken meat across all retail outlets was 6.45 log_10_ CFU/cm², exceeding the recommended microbiological threshold of 5.0 log_10_ CFU/cm² as reported in the Commission of the European Communities guidelines. The highest average number of bacterial counts was found in meat samples from butcher shops (6.77 log_10_ CFU/cm²), followed by those from supermarkets (6.39 log_10_ CFU/cm²), and public wet markets (6.18 log_10_ CFU/cm²). As illustrated in the boxplot, median TVC values exceeded acceptable microbiological limits across all value-chain sources (Figure 3). Although butcher shops exhibited slightly higher median counts and a narrower interquartile range compared with supermarkets and public wet markets, substantial overlap in the distributions was observed. Consistent with this pattern, the Kruskal–Wallis test indicated no statistically significant differences in TVC among butcher shops, supermarkets, and public wet markets (p > 0.05).

**Figure 4:**
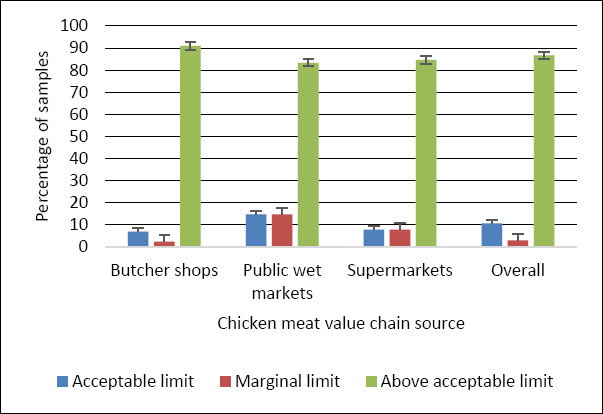
Proportion of chicken meat samples classified as acceptable limit [≤ 1 × 10^4^ CFU/cm^2^ (4.0 log)], marginal [≤ 1 × 10^5^ CFU/cm^2^ (5.0 log)] or above acceptable microbiological limits [> 1 × 10^5^ CFU/cm^2^ (5.0 log)] based on total viable bacterial counts across different value-chain sources. Each bar represents the percentage ± standard deviation (n = 105).

Classification of raw chicken meat samples based on TVC revealed a high-level of microbiological contamination across all value-chain sources (Figure 4). The majority of samples from butcher shops, supermarkets, and public wet markets were classified as above acceptable microbiological limits, accounting for more than 80% of samples in each category. Overall, 86.7% of samples surpassed the recommended limits, including 90.9% from butcher shops, 84.6% from supermarkets, and 83.3% from public wet markets. Only a small proportion of samples fell within the marginal range, while those meeting acceptable standards were rare across all outlets.

### Enumeration of TVC in pork samples

**Figure 5:**
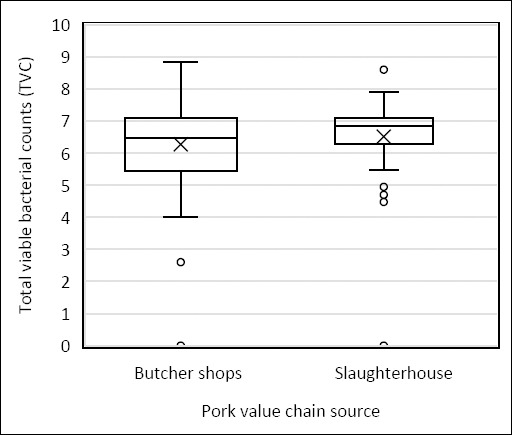
Distribution of total viable bacterial counts (log_10_ CFU/cm²) in raw pork obtained from butcher shops and slaughterhouses in Murang’a County, Kenya. The central line represents the median, boxes indicate the interquartile range, and whiskers represent the minimum and maximum values (n = 215; outliers are represented as circles).

A total of 215 pork samples were analyzed, comprising 164 from the butcher shops and 51 from the slaughterhouses. The overall mean TVC in pork meat samples was 6.39 log_10_ CFU/cm², which exceeds the recommended microbiological threshold of 5.0 log_10_ CFU/cm². The highest mean viable bacterial counts varied across value-chain sources, with generally higher levels recorded in pork samples from slaughterhouses (6.51 log_10_ CFU/cm²) than in butcher shops (6.27 log_10_ CFU/cm²), but no statistically significant differences were observed between sources (p > 0.05). As illustrated in the boxplot, the median TVC for slaughterhouse samples was higher, and the distribution was skewed toward elevated bacterial loads, indicating substantial contamination at the point of primary processing (Figure 5).

**Figure 6:**
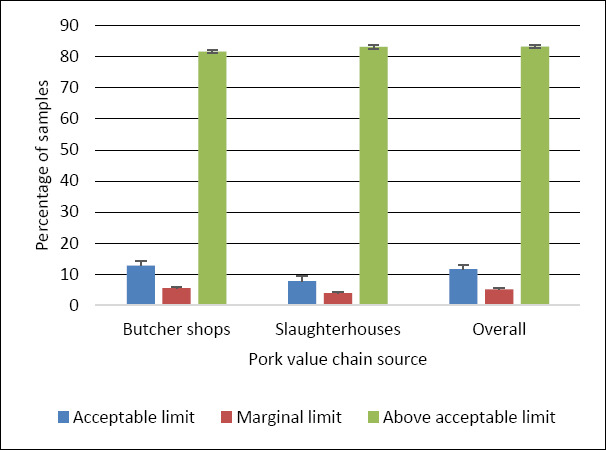
Proportion of raw pork samples classified as acceptable limit [≤ 1 × 10^4^ CFU/cm^2^ (4.0 log)], marginal [≤ 1 × 10^5^ CFU/cm^2^ (5.0 log)] or above acceptable microbiological limits [> 1 × 10^5^ CFU/cm^2^ (5.0 log)] based on total viable bacterial counts across different value-chain sources. Each bar represents the percentage ± standard deviation (n = 215).

Classification of raw pork samples based on TVC revealed a high-level of microbiological contamination across all value-chain sources (Figure 6). The majority of samples from butcher shops, and slaughterhouses were classified as above acceptable microbiological limits, accounting for more than 80% of samples in each category. Overall, 83.3% of pork samples exceeded acceptable limits, with comparable proportions observed in slaughterhouses (83.2%) and butcher shops (81.7%). Only a small proportion (16.7%) of pork samples across all sources were within marginal limits or below acceptable limits.

### Prevalence of *E. coli* and *Salmonella* species

The prevalence of *E. coli* and *Salmonella* spp. varied across sources in the chicken and pork value chains (Table 2). Overall, *E. coli* prevalence in chicken and pork samples was 61.6% (95% CI: 56.1–66.7), indicating high contamination across outlets. Prevalence was higher in pork (63.7%, 95% CI: 57.1–69.9) than in chicken (57.1%, 95% CI: 47.6–66.2), though overlapping confidence intervals indicate this difference was not statistically significant. In chicken retail outlets, contamination levels were similar, with wet markets highest at 60.4% (Table 4). Pork from slaughterhouses had higher *E. coli* prevalence (72.5%, 95% CI: 59.1–82.9) than pork from butcher shops (60.6%, 95% CI: 53.3–68.1) (p = 0.082).

**Table 2:** Prevalence of *Escherichia coli* and *Salmonella* across the different chicken and pork slaughterhouses and retail outlets.

Sample type and value-chain sources	*Escherichia coli*		*Salmonella*	

	Number of positive samples (n)	Prevalence (95% CI)	Number of positive samples (n)	Prevalence (95% CI)
Chicken meat sample				
Butcher shops (n = 44)	24	54.5% (40.1–68.3)	5	11.4% (5.0–24.0)
Supermarkets (n = 13)	7	53.8% (29.1–76.8)	0	0.0 % (0.0–22.8)
Public wet markets (n = 48)	29	60.4% (46.3–73.0)	1	2.1% (0.4–10.9)
Subtotal (n = 105)	60	57.1% (47.6–66.2)	6	5.7% (2.6–11.9)
Pork samples				
Butcher shops (n = 164)	100	60.6% (53.3–68.1)	16	9.8% (6.1–15.3)
Slaughterhouses (n = 51)	37	72.5% (59.1–82.9)	1	2.0% (0.3–10.3)
Subtotal (n = 215)	137	63.7% (57.1–69.9)	17	7.9% (5.0–12.3)
Total (N = 320)	197	61.6% (56.1–66.7)	23	7.2% (4.8–10.6)

**Table 3:** Prevalence of antimicrobial-resistant *Escherichia coli* isolates from chicken and pork samples obtained from slaughterhouses and retail outlets in Murang’a County, Kenya.

Antibiotic agent	Chicken meat samples				Pork samples			Overall total (n = 150)	

	Butcher shops (n = 15)	Supermarket (n = 5)	Public wet markets (n = 24)	Subtotal (n = 44)	Butcher shops (n = 81)	Slaughter houses (n = 25)	Subtotal (n = 106)	% (n)	Clopper–Pearson 95% CI
Ampicillin	100.0	100.0	91.7	95.5	95.1	96.0	95.3	95.3 (143)	90.6–98.1
Amoxicillin–clavulanate	93.3	60.0	70.8	77.3	79.0	80.0	79.2	78.7 (118)	71.2–84.9
Ceftriaxone	26.7	0.0	29.2	25.0	27.2	16.0	24.5	24.7 (37)	18.0–32.4
Ceftazidime	66.7	20.0	62.5	59.1	55.6	60.0	56.6	57.3 (86)	49.0–65.4
Imipenem	6.7	20.0	4.2	6.8	3.7	12.0	5.7	6.0 (9)	2.8–11.1
Gentamicin	86.7	80.0	66.7	75.0	71.6	72.0	71.7	72.7 (109)	64.8–79.6
Tetracycline	80.0	80.0	91.7	86.4	69.1	56.0	66.0	72.0 (108)	64.1–79.0
Ciprofloxacin	60.0	60.0	66.7	63.6	40.7	60.0	45.3	50.7 (76)	42.4–58.9
Trimethoprim-Sulfamethoxazole	73.3	80.0	79.2	77.3	55.6	60.0	56.6	62.7 (94)	54.4–70.4
Chloramphenicol	20.0	0.0	25.0	20.5	27.2	24.0	26.4	24.7 (37)	18.0–32.4

**Table 4:** Distribution of multiple antibiotic resistance indices and resistance categories among *Escherichia coli* isolates.

MAR index	Number of antimicrobials to which the isolates were resistant	Number of antimicrobial classes to which the isolates were resistant	Percentage of antimicrobial-resistant *E. coli* isolates from different sources, (n)			Character of resistant strains

			Pork (n = 106)	Chicken meat (n = 44)	Total (n = 150)	
0.1	1	1	1.9 (2)	4.5 (2)	2.7 (4)	Non-MDR
0.2	2	1	1.9 (2)	0.0 (0)	1.3 (2)	
		2	5.7 (6)	0.0 (0)	4.0 (6)	
0.3	3	2	6.6 (7)	0.0 (0)	4.7 (7)	
		3	6.6 (7)	2.3 (1)	5.3 (8)	MDR
0.4	4	3	9.4 (10)	13.6 (6)	10.7 (16)	
		4	3.8 (4)	2.3 (1)	3.3 (5)	
0.5	5	3	0.9 (1)	0.0 (0)	0.7 (1)	
		4	13.2 (14)	9.1 (4)	12.0 (18)	
		5	4.7 (5)	9.1 (4)	6.0 (9)	
0.6	6	4	1.9 (2)	2.3 (1)	2.0 (3)	
		5	11.3 (12)	15.9 (7)	12.7 (19)	
		6	0.9 (1)	0.0 (0)	0.7 (1)	
0.7	7	5	1.9 (2)	4.5 (2)	2.7 (4)	
		6	14.2 (15)	15.9 (7)	14.7 (22)	
		7	0.0 (0)	2.3 (1)	0.7 (1)	XDR
0.8	8	6	4.7 (5)	4.5 (2)	4.7 (7)	
		7	5.7 (6)	6.8 (3)	6.0 (9)	
0.9	9	7	2.8 (3)	4.5 (2)	3.3 (5)	
		8	0.9 (1)	2.3 (1)	1.3 (2)	
1.0	10	8	0.9 (1)	0.0 (0)	0.7 (1)	PDR

MAR = Multiple antibiotic resistance, MDR = Multidrug-resistant, XDR = Extensively drug-resistant, PDR = Pan drug-resistant.

Table 2 shows a relatively low overall *Salmonella* prevalence of 7.2% (95% CI: 4.8%–10.6%). In chicken, prevalence was 5.7% (95% CI: 2.6%–11.9%), with no positive isolates from supermarkets. Pork had a slightly higher prevalence of 7.9% (95% CI: 5.0%–12.3%). Despite these differences, they were not statistically significant (p = 0.32). Most *Salmonella*-positive pork samples were from butcher shops (9.8%, 16/164), while detection in slaughterhouses was rare (2.0%, 1/51).

### Antimicrobial susceptibility profiles of *E. coli* isolates

Table 3 summarizes the antimicrobial susceptibility patterns of *E. coli* isolates recovered from raw chicken meat (n = 44) and pork (n = 106) samples. Overall, the highest resistance levels were observed in AMP (95.3%, 95% CI: 90.6-98.1) and AMC (78.7%, 95% CI: 71.2-84.9). Elevated resistance rates were also detected for CN (72.7%, 95% CI: 64.8-79.6), TET (72.0%, 95% CI: 64.1-79.0), STX (62.7%, 95% CI: 54.4-70.4), CAZ (57.3%, 95% CI: 49.0-65.4), and CIP (50.7%, 95% CI: 42.4-58.9). Moderate resistance levels were noted for CRO and C each at 24.7% (95% CI: 18.0-32.4), whereas the lowest resistance prevalence was recorded for IMP at 6.0% (95% CI: 2.8-11.1).

When stratified by meat type, *E. coli* isolates from chicken meat exhibited a higher prevalence of resistance to TET, CIP, STX, and CAZ than isolates from pork. By contrast, resistance to AMP was uniformly high in isolates from both meat types and across all retail sources, with proportions ranging from 91.7% to 100%. Resistance to IMP remained low across most source categories, although slightly elevated proportions were observed among pork-derived isolates from slaughterhouses (12.0%) and chicken-derived isolates from supermarkets (20.0%). This finding should be interpreted cautiously, given the limited sample sizes in these subgroups.

### MAR indices and resistance patterns among *E. coli* isolates

**Figure 7:**
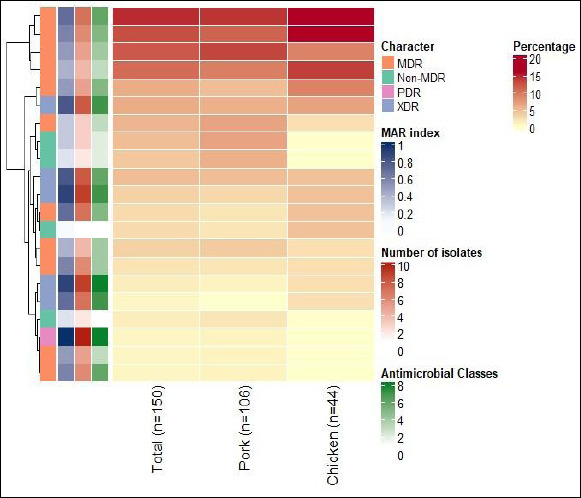
The heatmap shows antimicrobial resistance patterns across meat samples. Categorical isolate types, MDR, XDR, PDR, and Non-MDR, are indicated with distinct color blocks. Percentages use a yellow-to-red gradient (0–25%), with darker red indicating higher values. The MAR index (0–1) is shown on a blue gradient, where darker blue indicates higher resistance. Isolate counts (0–10) use a purple gradient, and the number of antimicrobial classes resisted (0–8) uses a green gradient, with darker shades indicating higher values. In all cases, greater color intensity reflects a higher magnitude of the variable, enabling quick visual comparison.

MDR was observed in 87.3% (131/150; 95% CI: 81.1-91.7%) of *E. coli* isolates, with only 12.7% (19/150; 95% CI: 8.3-18.9%) classified as non-MDR. XDR isolates accounted for 16.0% (24/150; 95% CI: 10.7-22.9%) and were susceptible to two or fewer antimicrobial classes. Only one isolate (0.7%; 1/150; 95% CI: 0.1–3.7%) exhibited resistance to all tested antimicrobial agents and was classified as possible PDR. The heatmap (Figure 7) illustrates the distribution of MAR indices and resistance categories (MDR, XDR, and PDR) among *E. coli* isolates across different meat samples.

Determination of the MAR indices revealed that all tested pork isolates had MAR indices≥0.1, and chicken isolates similarly exhibited MAR indices ≥0.1, indicating exposure to antimicrobial agents and suggesting that both meat sources represent potential high-risk reservoirs of antimicrobial-resistant *E. coli* (Table 4). Notably, 34.0% (51/150; 95% CI: 26.9–41.9%) *E. coli* isolates exhibited MAR indices ≥0.7 (corresponding to resistance to seven or more antimicrobials). Of these, 33 (31.1%) were recovered from pork samples and 18 (40.9%) from chicken samples (Table 4), demonstrating a substantial proportion of extensively resistant strains in both meat types.

A wide range of MDR phenotypes was observed among *E. coli* isolates, indicating extensive heterogeneity in resistance profiles. The most common pattern was AMP–AMC–CAZ–CN–TET–CIP–SXT, reflecting concurrent resistance to AMP, AMC, CAZ, CN, TET, CIP, and STX. Variants of this core β-lactam–aminoglycoside–fluoroquinolone–tetracycline–folate resistance profile were repeatedly detected. High-level combinations involving third-generation cephalosporins (CAZ and CRO) with CIP, CN, and TET were common. Some isolates also showed IMP resistance, yielding extensive profiles such as AMP–AMC–CRO–CAZ–IMP–CN–TET–CIP–SXT, spanning nearly all tested antimicrobial classes. Lower-level patterns (e.g., AMP alone; AMP–AMC; AMP–CN; TET alone) were less frequent than complex MDR combinations.

### AMR profiles of *Salmonella* isolates

AMR patterns of *Salmonella* isolates varied according to both antibiotic agent and sample type, as illustrated in Table 5. Overall, CIP showed the highest resistance (86.4%, 95% CI: 65.1-97.1), with all pork-derived isolates (100%) and half of chicken-derived isolates (50.0%) exhibiting resistance. AMP resistance was 31.8% (95% CI: 13.9-54.9) overall, higher in chicken (66.7%) than in pork (18.8%). CRO resistance occurred in 18.2% (95% CI: 5.2-40.3) of isolates, mainly from chicken (50.0%), while TET resistance (13.6%, 95% CI: 4.8–33.4%) was confined to pork isolates. AZI resistance was low (4.5%, 95% CI: 0.1-22.8) and was observed only in pork isolates. Zero resistance was observed to IMP, STX, or C.

**Table 5:** Antimicrobial resistance patterns of *Salmonella* isolates from chicken meat and pork samples from Murang’a County, Kenya.

Antibiotic agent	Chicken meat samples			Pork samples			Overall total (n = 22)	

	Butcher shops (n = 5)	Public wet markets (n =1)	Subtotal (n = 6)	Butcher shops (n = 15)	Slaughterhouses (n = 1)	Subtotal (n = 16)	% (n)	Clopper–pearson 95% CI
Ampicillin	60.0	100.0	66.7	13.3	100.0	18.8	31.8 (7)	13.9-54.9
Ceftriaxone	40.0	100.0	50.0	6.7	0.0	6.3	18.2 (4)	5.2-40.3
Azithromycin	0.0	0.0	0.0	6.7	0.0	6.3	4.5 (1)	0.1-22.8
Imipenem	0.0	0.0	0.0	0.0	0.0	0.0	0.0 (0)	0.0-15.4
Tetracycline	0.0	0.0	0.0	20	0.0	18.8	13.6 (3)	2.9-34.9
Ciprofloxacin	40.0	100.0	50.0	100	100.0	100.0	86.4 (19)	65.1-97.1
Trimethoprim-sulfamethoxazole	0.0	0.0	0.0	0.0	0.0	0.0	0.0 (0)	0.0-15.4
Chloramphenicol	0.0	0.0	0.0	0.0	0.0	0.0	0.0 (0)	0.0-15.4

CI = Confidence interval.

### MAR indices and resistance patterns among *Salmonella* isolates

The distribution of MAR indices and resistance patterns among the *Salmonella* isolates is presented in Table 6. MAR index analysis of *Salmonella* isolates showed varying resistance in pork and chicken meat. One chicken isolate (4.5%) was fully susceptible (MAR = 0.00). Single-drug resistance (MAR = 0.13) was primarily attributed to CIP resistance and accounted for the largest proportion of the isolates. Resistance to two antimicrobial classes (MAR = 0.25) occurred in 31.8% of isolates, with common patterns TET–CIP (18.8% in pork), AMP-CIP (12.5% in pork), CRO-CIP (6.3% in pork), and AMP-CRO (16.7% in chicken). MDR (MAR = 0.38) was found in 13.6% of isolates, mainly AMP-AZI-CIP (6.3% in pork) and AMP-CRO-CIP (33.3% in chicken). MDR isolates in both pork and chicken highlight the presence of resistant *Salmonella* in retail meat.

**Table 6:** Distribution of multiple antibiotic resistance indices and resistance patterns among *Salmonella* isolates from different sources.

MAR index	Number of antimicrobials to which the isolates were resistant	Resistance pattern	Number of resistant *Salmonella* isolates from different sources (%)			Character of resistant strains

			Pork (n = 16)	Chicken meat (n = 6)	Total (n = 22)	
0.00	0	-	0 (0)	1 (16.7)	1 (4.5)	Non-MDR
0.13	1	AMP	0 (0)	1 (16.7)	11 (50)	
		CIP	10 (62.5%)	0 (0)		
0.25	2	CRO-CIP	1 (6.3)	0 (0)	7 (31.8)	
		AMP-CRO	0 (0)	1 (16.7)		
		AMP-CIP	2 (12.5)	0 (0)		
		TET–CIP	3 (18.8)	0 (0)		
0.38	3	AMP-AZI-CIP	1 (6.3)	0 (0)	3 (13.6)	MDR
		AMP-CRO-CIP	0 (0)	2 (33.3)		

AMP = Ampicillin; CRO = Ceftriaxone; TET = Tetracycline; AZI = Azithromycin; CIP = Ciprofloxacin; MDR = Multidrug resistance.

## DISCUSSION

### Bacterial contamination and TVC in meat

This study analyzed bacterial contamination and the prevalence of resistant *E. coli* and *Salmonella* in raw chicken meat and pork from slaughterhouses and market outlets in Central Kenya. TVC indicate the level of bacterial contamination in meat and its suitability for human consumption. The interpretation of TVC in this study was guided by internationally recognized benchmarks commonly applied in meat hygiene assessment, including the 5.0 log_10_ CFU/cm² threshold frequently referenced in European process hygiene criteria (e.g., Commission Regulation (EC) No. 2073/2005) [47]. While such limits provide a useful framework for evaluating microbial quality and processing conditions, it is important to note that neither Codex Alimentarius standards nor national regulations in many African countries, including Kenya, define strict regulatory limits for TVC in raw meat. Therefore, the threshold used in this study should be understood as an internationally cited reference for hygienic assessment rather than a legally binding national standard. This contextualization supports interpretation of findings within a global food safety framework.

The current study provides evidence that raw meat from various slaughterhouses and retail markets in Murang’a County, Kenya, is highly contaminated with bacteria. Comparable findings have been reported in several sub-Saharan African settings, where suboptimal hygienic practices, inadequate sanitation infrastructure, and informal or poorly regulated processing systems frequently permit elevated microbial loads to be maintained in retail meat products [36, 46, 53]. Conversely, lower results were reported in other studies: 4.03 log CFU/g in Uganda [54], and 2.159–2.736 log CFU/g in Kenya [55]. The discrepancies between this study and previous ones may be attributable to differences in meat type and source, hygiene conditions, and study settings. Additionally, the consistently high contamination levels observed across butcher shops, supermarkets, slaughterhouses, and wet markets indicate that this problem is not confined to any one type of outlet. Rather, it points to deeper, system-wide gaps in how meat is handled, processed, and sold. This highlights the need for integrated, collaborative efforts across the entire meat value chain to strengthen hygiene and surveillance in the study region [30].

Studies from Ethiopia, South Africa, and Malaysia have documented comparable patterns, particularly in contexts where cold-chain infrastructure is unreliable or where cleaning and sanitation protocols are inconsistently implemented [35, 56]. In the current study, statistically insignificant variations in TVC across most slaughter and retail outlet sources corroborate the hypothesis that microbial contamination risks are cumulative and evident throughout the continuum from slaughter to retail. These findings underscore the necessity of targeted, context-appropriate interventions, including enhanced personnel training, rigorous enforcement of hygiene regulations, and systematic implementation of structured food safety frameworks such as Good Hygienic Practices and Hazard Analysis and Critical Control Points [15, 32].

### Prevalence of *E. coli* and *Salmonella* contamination

The high prevalence of *E. coli* (61.6%) in this study provides further evidence of extensive fecal contamination throughout the meat value chain. This finding is consistent with global reports, as *E. coli* is widely used as a sentinel indicator of hygienic failures in meat processing environments [16, 57]. The proportion of *E. coli*-positive samples in this study was significantly lower than that reported in similar studies in other parts of Nairobi, Kenya: 78% [58] and 72% [32]. The slightly higher *E. coli* contamination rate in pork (63.7%) compared with chicken meat (57.1%) may reflect species-specific differences in slaughter procedures, carcass handling, or process hygiene [30]. Particularly noteworthy in this study is the significantly higher occurrence of *E. coli* in slaughterhouses than in butcher shops, suggesting that contamination likely originates earlier in processing, especially during slaughter and carcass dressing, as indicated by previous findings from Eng *et al*. [59] and FAO/WHO [35]. Other studies in Kenya have indicated that there is generally low hygiene knowledge and practice in slaughterhouses and among slaughter workers [29, 60].

Although the overall prevalence of *Salmonella* was relatively low (7.2%), its occurrence represents a significant public health concern. Higher prevalence of *Salmonella* (17–18%) in meat has been reported in Kenya [29]. Non-typhoidal *Salmonella* remains one of the major foodborne diseases associated with the highest mortality globally [27, 61]. The marginally higher contamination rate observed in pork (7.9%) compared with chicken (5.7%) aligns with reports from Kenya [28] and other African meat production and retail systems [62, 63]. Furthermore, the predominance of positive samples originating from butcher shops suggests that cross-contamination may occur via food handlers, processing equipment, or contact surfaces [32]. The absence of *Salmonella* in chicken from supermarkets may reflect more effective cold-chain maintenance and stricter adherence to hygienic practices compared with less-regulated, open retail settings.

### AMR patterns in *E. coli* and *Salmonella*

The high AMR rates among *E. coli* and *Salmonella* isolates were reported in this study, highlighting significant emerging severe threats to public health and complicating effective therapeutic management. The high levels of resistance in *E. coli*, particularly to AMP (95.3%), AMC (78.7%), TET (72.0%), STX (62.7%), and CN (72.7%), are consistent with other findings in similar studies reported in Kenya and other regions [28, 37, 64, 65]. Many of these antimicrobials are widely used in livestock production in Kenya [26, 39], often under limited regulatory oversight, which likely facilitates the selection, amplification, and dissemination of resistant bacterial populations [66, 67].

*E. coli* isolates showed significant resistance to CIP (50.7%). This was significantly higher than what was reported in previous studies on meat in Kenya. For instance, Muinde *et al*. [28] in their study in Nairobi reported a prevalence of 6%–7% in raw pork and poultry meat from major retail chains. Similarly, Odwar *et al*. [58] reported a prevalence of 4.5% in raw chicken retail meat in Nairobi. This high resistance reported in this study is particularly concerning and suggests an alarming shift to fluoroquinolone resistance, especially in the meat value chain. To preserve this critically important antimicrobial for human medicine, further studies are necessary to determine its source [68].

Notable resistance to third-generation cephalosporins, including CRO (24.7%) and CAZ (57.3%), was observed in this study, with similar trends for both chicken and pork. These findings strongly indicate the potential of extended-spectrum β-lactamase-producing *E. coli*. Similar prevalence of extended-spectrum β-lactamase-producing bacteria has been reported in Thailand in pork (46%) and chicken meat (69.7%) from fresh markets [69]. However, previous studies have reported a lower prevalence of extended-spectrum β-lactamases in Kenya from meat of about 6.3% from slaughterhouses [30] (5%–11%) from retail markets [28]. Extended-spectrum β-lactamase strains are MDR, clinically challenging to treat, and hence classified under WHO priority pathogens [8, 68]. The high level of cephalosporins reported in this study could be due to cross-contamination of the meat from other reservoirs, since third-generation cephalosporins are restricted for use in poultry and pigs in Kenya, as in other countries globally [70]. However, the study findings warrant further investigation to determine the source of the resistance in meat.

IMP exhibited the lowest resistance rate among all antibiotics tested, at 6% in *E. coli* and 0% in *Salmonella* isolates. This low resistance is expected, as IMP is a carbapenem classified by the WHO as a highest-priority critically important antimicrobial for human medicine. However, slightly lower resistance (2%) has been reported among *E. coli* isolates recovered from raw meat sold in Nairobi, Kenya [71], while a marginally higher resistance rate (2.5%) in *Salmonella* was documented in broiler chickens in Uganda [72]. Although the differences are small, these findings suggest regional variability in carbapenem resistance patterns across East African food production systems. Carbapenems are generally reserved for severe hospital infections and are not approved for routine use in food-producing animals in many countries, including Kenya. Consequently, limited use in livestock likely explains the largely preserved susceptibility observed. Metagenomic analyses of cattle from Kenya, Uganda, and Tanzania have detected AMR genes associated with resistance to critical drug classes, including carbapenems, suggesting that genetic reservoirs for carbapenem resistance are already present within livestock populations, even where phenotypic resistance in food products remains infrequently reported [73]. Furthermore, the detection of carbapenem-resistant Enterobacterales in food matrixes raises concerns about possible contamination from human reservoirs, environmental sources (e.g., wastewater or manure), or unauthorized antimicrobial use in animal production systems [34, 68]. Although detection in food remains relatively infrequent globally, emerging data indicate an increasing occurrence [74]. This study therefore provides some of the earliest evidence of emerging carbapenem resistance within the food chain in Kenya. More studies are needed to determine the source and resistance mechanism [25].

### MDR and MAR index implications

The high frequency of MDR observed among food-chain *E. coli* isolates indicates substantial antimicrobial selection pressure within the production and retail continuum. This interpretation is further supported by the high MAR index values (≥0.5), which are indicative of exposure to high-risk sources of contamination [37]. The observed MAR distribution suggests that pork and chicken production systems face substantial antimicrobial pressure, likely from therapeutic, prophylactic, or growth-promoting use. The presence of MDR, XDR, and PDR phenotypes in the food chain underscores the urgent need to strengthen antimicrobial stewardship in food animal production, conduct routine resistance surveillance, and implement hygiene interventions at slaughter and retail.

The predominance of complex resistance combinations, particularly involving β-lactams (AMP, AMC, CAZ, CRO), aminoglycosides (CN), fluoroquinolones (CIP), tetracyclines (TET), and trimethoprim-sulfamethoxazole (STX), indicates sustained antimicrobial selection pressure within the meat production continuum. Notably, the dominant resistance profile spans multiple major antibiotic classes commonly used in livestock production systems [26, 75]. Co-resistance to β-lactams, tetracycline, sulfonamides, and fluoroquinolones suggests plasmid-mediated determinants driving horizontal transfer and persistence. Detection of isolates resistant to nearly all tested drugs, including carbapenems, is especially alarming. Although carbapenems are rarely used in veterinary practice, resistance in foodborne isolates may arise from environmental spread of carbapenemase genes or indirect selection via co-resistance. These widespread MDR patterns indicate that retail meat is an important reservoir of resistant bacteria and genes, underscoring the need for stricter antimicrobial stewardship in livestock, routine extended-spectrum β-lactamase and carbapenemase surveillance, and enhanced hygiene throughout the meat value chain.

### Public health implications and One Health perspective

Murang’a shows a distinct AMR profile, with higher carbapenem resistance in rural isolates than in urban centers such as Nairobi, where most national surveillance occurs, suggesting underrecognized reservoirs of MDR pathogens likely driven by limited healthcare access, over-the-counter antibiotic use, and close human–livestock contact. These resistance patterns, coupled with microbial contamination along the meat value-chain, where poor hygiene spreads resistant strains and unregulated antimicrobial use in animal production increases selective pressure, underscore the county’s underrepresentation in national and global databases and position Murang’a as a key sentinel site for early detection of antimicrobial resistance. Together, these challenges highlight the urgent need for a One Health–based AMR mitigation strategy in Murang’a County and across Kenya.

### Study limitations and future directions

Nevertheless, the study has certain limitations. The purposive selection of sub-counties may introduce selection bias and limit generalizability. Although samples were randomly selected within outlets, inclusion depended on product availability at the time of visit, potentially causing availability bias. Vendor participation based on verbal consent, while suitable for minimal-risk observational studies, may have affected responses. However, the inclusion of diverse retail categories and wide geographic coverage strengthens the dataset. MALDI-TOF MS was employed in this study for species-level identification of the isolates. However, this technique does not provide a reliable resolution for determining *Salmonella* serovars or *E. coli* serotypes. The absence of serological or molecular typing methods (e.g., slide agglutination, PCR-based serotyping, or whole-genome sequencing) limits the ability to infer strain diversity and epidemiological linkages. The study focused exclusively on raw meat samples and did not include environmental, human, or animal-farm comparison samples. As a result, potential transmission pathways and links between food, humans, and the environment could not be assessed. Additionally, no molecular analyses were performed to identify specific resistance genes or mechanisms. Consequently, the genetic basis of the observed resistance patterns could not be determined.

Future studies should comprehensively characterize circulating strains using serotyping and genomics to identify virulence factors, resistance mechanisms, and genetic diversity. Routine molecular surveillance would enable early detection and tracking of emerging or resistant strains. Investigating contextual risk factors, such as hygiene practices, animal husbandry, and antimicrobial use, together with longitudinal monitoring, would clarify temporal trends, persistence, and transmission dynamics.

Adopting a One Health framework with integrated sampling across animal, human, and environmental interfaces is essential. Expanding surveillance to slaughterhouse workers (e.g., hand or fecal samples), market vendors, and nearby environmental sources such as water would enable comparison of AMR profiles and, where possible, genomic relatedness between sectors. This integrated approach would clarify transmission and spillover dynamics, deepen understanding of pathogen ecology, and support evidence-based control strategies. It could also make future study models for holistic AMR surveillance in rural settings in developing countries.

## CONCLUSION

This study demonstrated a high level of bacterial contamination in raw chicken meat and pork across slaughterhouses and retail outlets in Murang’a County, Kenya, as evidenced by TVC values exceeding internationally accepted hygienic thresholds. The findings further revealed a high prevalence of *E. coli* (61.6%) and a detectable presence of *Salmonella* (7.2%), confirming widespread fecal contamination and potential food safety risks across the meat value chain. Antimicrobial susceptibility testing showed substantial resistance among *E. coli* isolates, particularly to AMP, AMC, TET, STX, CN, and CIP, with notable resistance also observed to third-generation cephalosporins (CRO and CAZ). Although resistance to IMP remained low, its detection highlights emerging concerns regarding critically important antimicrobials. The predominance of MDR (87.3%) in *E. coli*, alongside the occurrence of XDR and PDR phenotypes and consistently high MAR index values, further indicates strong antimicrobial selection pressure within the production and retail continuum.

From a practical perspective, these findings underscore the urgent need for strengthening hygiene and sanitation practices across all stages of the meat value-chain, particularly at slaughter, public wet markets, and retail levels where contamination appears to originate and persist. Implementation of structured food safety systems, including Good Hygienic Practices and Hazard Analysis and Critical Control Points, should be prioritized. In addition, stricter regulation and stewardship of antimicrobial use in livestock production are essential to limit the emergence and spread of resistant bacteria. Routine microbiological monitoring and AMR surveillance should be integrated into national food safety frameworks, supported by a One Health approach that considers human, animal, and environmental interfaces.

A key strength of this study lies in its comprehensive assessment of microbial contamination and AMR across multiple value chain nodes, including slaughterhouses, butcher shops, supermarkets, and public wet markets. The use of MALDI-TOF MS for bacterial identification enhances the reliability of species-level characterization, while the inclusion of MAR index analysis provides deeper insight into resistance pressure and epidemiological risk. Furthermore, the study offers important baseline data from a region that is currently underrepresented in national and global AMR surveillance systems.

In conclusion, raw meat sold in Murang’a County represents a significant reservoir of antimicrobial-resistant bacteria, posing a potential risk to public health. The convergence of high microbial loads, widespread AMR, and evidence of multidrug-resistant phenotypes highlights the need for coordinated interventions. Strengthening hygiene practices, enforcing antimicrobial stewardship, and expanding integrated surveillance systems are critical steps toward mitigating AMR and improving food safety in Kenya and similar settings.

## DATA AVAILABILITY

The data supporting the findings of this study are available from the corresponding author upon reasonable request.

## AUTHORS’ CONTRIBUTIONS

MNN, CMM, DWW, GSP, GCG, JNO, and CDK: Conceptualization and study design. MNN, CMM, and DWW: Laboratory work. FMK and DWW: Data analysis and interpretation. MNN, CMM, DWW, GSP, GCG, JNO, CDK, and FMK: Interpretation of results and writing—original draft and manuscript preparation. All authors have read, reviewed, and approved the final manuscript.
